# NEAr Transporter (NEAT) Domains: Unique Surface Displayed Heme Chaperones That Enable Gram-Positive Bacteria to Capture Heme-Iron From Hemoglobin

**DOI:** 10.3389/fmicb.2020.607679

**Published:** 2021-01-06

**Authors:** Ken Ellis-Guardiola, Brendan J. Mahoney, Robert T. Clubb

**Affiliations:** ^1^Department of Chemistry and Biochemistry, University of California, Los Angeles, Los Angeles, CA, United States; ^2^UCLA-DOE Institute for Genomics and Proteomics, University of California, Los Angeles, Los Angeles, CA, United States; ^3^Molecular Biology Institute, University of California, Los Angeles, Los Angeles, CA, United States

**Keywords:** *Staphylococcus aureus*, heme, hemoglobin, NEAr transporter domains, sortase, iron regulated surface determinant system, iron, pathogen

## Abstract

Iron is an important micronutrient that is required by bacteria to proliferate and to cause disease. Many bacterial pathogens forage iron from human hemoglobin (Hb) during infections, which contains this metal within heme (iron–protoporphyrin IX). Several clinically important pathogenic species within the Firmicutes phylum scavenge heme using surface-displayed or secreted NEAr Transporter (NEAT) domains. In this review, we discuss how these versatile proteins function in the *Staphylococcus aureus* Iron-regulated surface determinant system that scavenges heme-iron from Hb. *S. aureus* NEAT domains function as either Hb receptors or as heme-binding chaperones. *In vitro* studies have shown that heme-binding NEAT domains can rapidly exchange heme amongst one another via transiently forming transfer complexes, leading to the interesting hypothesis that they may form a protein-wire within the peptidoglycan layer through which heme flows from the microbial surface to the membrane. In Hb receptors, recent studies have revealed how dedicated heme- and Hb-binding NEAT domains function synergistically to extract Hb’s heme molecules, and how receptor binding to the Hb-haptoglobin complex may block its clearance by macrophages, prolonging microbial access to Hb’s iron. The functions of NEAT domains in other Gram-positive bacteria are also reviewed.

## Introduction

Nearly all bacterial pathogens require iron to grow because it is an essential metal cofactor that is used by microbial enzymes to mediate cellular metabolism. Its power in biology stems from its ability to toggle between Fe^*II*^ (ferrous) and Fe^*III*^ (ferric) oxidation states, with this redox activity playing key roles in metabolic enzymes (oxidases, catalases, peroxidases), electron transfer (iron-sulfur proteins, cytochromes), and DNA synthesis (ribonucleotide reductases). During infections, many pathogens forage iron from human hemoglobin (Hb), as it contains ∼ 75–80% of the human body’s total iron content in the form of heme (iron-protoporphyrin IX) (Parrow et al., 2013; Contreras et al., 2014; Wandersman and Delepelaire, 2014; Ma et al., 2015; Sheldon and Heinrichs, 2015; Choby and Skaar, 2016; Sheldon et al., 2016; Huang and Wilks, 2017; Conroy et al., 2019). Bacteria gain access to Hb after it is released from senescent erythrocytes that spontaneously lyse or when erythrocytes are actively lysed by bacterial cytotoxins. Dedicated bacterial import systems then scavenge the heme and degrade it to release free iron. These systems have important roles in bacterial pathogenesis and are therefore potential targets for new antimicrobial agents. Microbial import is challenging because heme is lipophilic, prone to aggregation via non-specific interactions, and it can generate damaging reactive oxygen species that are toxic to the cell (Huang and Wilks, 2017; Donegan et al., 2019). Here we review how Gram-positive bacteria within the Firmicutes phylum acquire heme using surface displayed or secreted NEAr Transporter (NEAT) domains (Andrade et al., 2002; Honsa et al., 2014).

**FIGURE 1:**
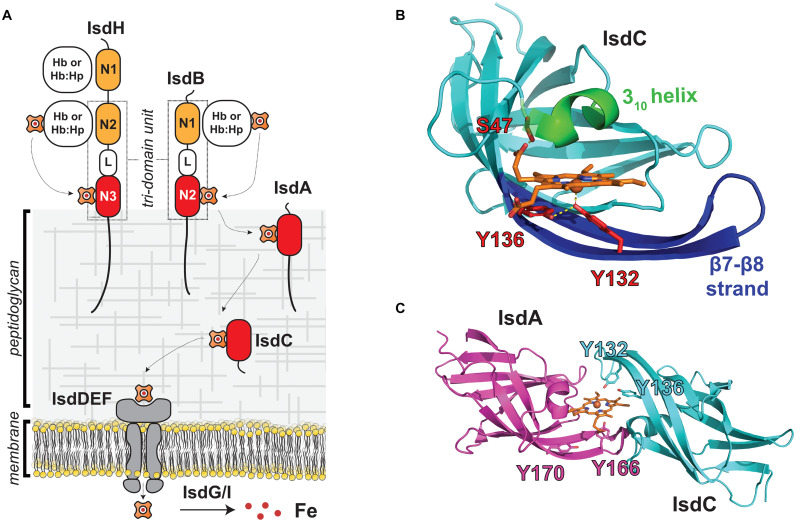
NEAT domains act as heme chaperones on the surface of *S. aureus*. **(A)** Schematic of the *S. aureus* Isd heme acquisition system that uses NEAT domains to bind heme and Hb. The IsdB and IsdH proteins are Hb receptors and contain multiple NEAT domains that bind to either heme (red) or Hb (orange) and are bridged by a helical linker (L) domain. NEAT domains in the IsdA and IsdC proteins shuttle heme across the cell wall for import into the cell where it is degraded. Proteins containing NEAT domains are attached to the cell wall by SrtA (IsdA, IsdB, IsdC, and IsdH) or SrtB (IsdC) sortase enzymes. **(B)** Structure of the IsdC NEAT domain bound to pentacoordinate heme [PDB: 2O6P, (Sharp et al., 2007)]. Heme-binding residues in the S/YXXXY motif are labeled in red, the conserved 3_10_ helix is indicated in green, and the β7/β8 strands are shown in blue. Important hydrogen bonding and axial coordination interactions shown with yellow dashes **(C)** NEAT domains in IsdA [purple, PDB: 2ITF, (Grigg et al., 2007)] and IsdC [cyan, PDB: 2O6P, (Sharp et al., 2007)] transiently associate with one another to rapidly exchange heme. Model of the heme “handclasp” complex consistent with NMR, mass spectrometry and X-ray crystallography data. Other heme-binding NEAT domains also associate with one another to rapidly transfer. Their unique ability to rapidly exchange heme suggests that NEAT domains within the cell wall form a protein wire that transfers heme from the cell surface to the membrane.

The function of NEAT domains in microbial heme scavenging is best understood for *Staphylococcus aureus*, which employs the Iron-regulated surface determinant (Isd) system to capture heme-iron from Hb (Figure 1A; Mazmanian et al., 2003; Nobles and Maresso, 2011; Sheldon and Heinrichs, 2015; Conroy et al., 2019). In the Isd system, four proteins containing NEAT domains are covalently attached to the cell wall (IsdA, IsdB, IsdC, and IsdH). IsdA and IsdC each possess a single NEAT domain that binds to heme with high affinity, while the IsdB and IsdH proteins contain multiple NEAT domains that bind to either heme (IsdB-N2, IsdH-N3) or Hb (IsdB-N1, IsdH-N1, IsdH-N2). The NEAT domain-containing Isd proteins are covalently attached to the cell wall by sortase enzymes, either by the housekeeping SrtA sortase (IsdA, IsdB, and IsdH), or by the SrtB sortase (IsdC) (Schneewind and Missiakas, 2012; Jacobitz et al., 2017). The Isd proteins are attached to the peptidoglycan at their C-termini via a peptide bond to the pentaglycine cell wall cross bridge. They are positioned at different depths within the cell wall as evidenced by their susceptibility to proteolytic degradation in whole cells. Their distinct locations are presumably dictated by differences in the number of amino acids that separate the last domain in each protein’s primary sequence to their cell wall attached C-termini, with proteins containing longer segments being positioned farther from the membrane (Mazmanian et al., 2003). Positional differences may also depend upon the type of sortase enzyme that is used to attach the protein to the cell wall, since unlike SrtA, the SrtB sortase attaches IsdC to uncrosslinked glycan strands (Marraffini and Schneewind, 2005). Finally, the cell wall locations of the Isd proteins may also depend upon their N-terminal signal peptide sequences, as some cell wall attached Isd proteins contain a YSIRK/GS motif within their signal peptides that primes them for anchoring to the cross wall that forms at the site of cell division (DeDent et al., 2008).

The cell wall-attached NEAT domains are thought to have distinct roles in the process of heme scavenging from Hb (Figure 1A). IsdB and IsdH are located toward the cell periphery to capture Hb and remove its heme molecules. Heme then moves to the partially embedded IsdA protein, and then to IsdC, which is located closest to the membrane. IsdC is believed to perform the final step in heme transfer through the cell wall by unloading its heme to IsdE, which is a component of a ATP-binding cassette (ABC)-transporter complex that pumps heme into the cell. Based on their primary sequences, the IsdE and IsdF proteins are believed to function as the ligand binding and permease components of the transporter. The IsdD protein has also been suggested to be part of the transporter complex, but its function is unclear as it does not share significant sequence homology with any protein of known function. The nucleotide binding domain (NBD) component of the transporter that powers heme import has also not been characterized; however, it is possible that FhuC performs this task as it is a promiscuous NBD that has been proposed to function broadly in iron uptake (Beasley et al., 2009). After transport across the membrane, heme is either directly incorporated into bacterial proteins or degraded by the IsdG or IsdI heme oxygenases to release free iron (Wu et al., 2005). Because excess heme is toxic, *S. aureus* also employs a HrtAB complex to export heme, and heme homeostasis is maintained by coordinating heme biosynthesis, import, and export functions (Torres et al., 2007; Stauff and Skaar, 2009). The general features of the Isd heme uptake system are conserved amongst other Firmicutes bacteria, which also employ NEAT domains to acquire heme.

A number of excellent reviews have been written that describe how bacteria acquire heme and employ it as an iron source (Runyen-Janecky, 2013; Contreras et al., 2014; Sheldon and Heinrichs, 2015; Choby and Skaar, 2016; Huang and Wilks, 2017). In this mini-review, we discuss what is known about the structural basis of NEAT domain function in *S. aureus*. We highlight the unique ability of these domains to rapidly exchange heme amongst one another and suggest that it may enable them to form a protein wire within the cell wall through which heme rapidly flows from the microbial surface to the membrane. We also discuss how variations in their primary sequences allow some NEAT domains in *S. aureus* to function as Hb receptors that can rapidly strip heme from Hb, and we briefly review what is known about the functions of NEAT domains in other species of Gram-positive bacteria.

## Heme-Binding NEAT Domains: Novel Chaperones That Rapidly Transfer Heme

Atomic structures of the heme-binding NEAT domains from *S. aureus* have been determined revealing a unique mode of ligand binding (IsdA, IsdB-N2, IsdC, and IsdH-N3) (Figure 1B; Grigg et al., 2007, 2011; Sharp et al., 2007; Villareal et al., 2008; Watanabe et al., 2008; Gaudin et al., 2011; Moriwaki et al., 2011; Vu et al., 2013). Structures of the IsdA- and IsdC-heme complexes were among the first to be determined, and revealed that NEAT domains adopt a conserved fold that binds heme within a hydrophobic cleft that is located at the end of its β-barrel structure (Grigg et al., 2007; Sharp et al., 2007). One face of heme’s protoporphyrin ring lies flat on the surface formed by residues in strands β7 and β8, whereas the other face is contacted by a “lip region” that forms a 3_10_ helix. Subsequent studies showed that heme-binding NEAT domains typically harbor a conserved serine and YXXXY sequence motifs that are located within the lip region and strand β8, respectively (hereafter called the S/YXXXY motif) (Honsa et al., 2014). The tyrosine residues are located in strand β8 and have important functions in the pentacoordinate ligation of heme iron (Figure 1B, red). The first tyrosine directly coordinates the metal (primary tyrosine), while the second (secondary tyrosine) forms a hydrogen bond to the primary tyrosine to stabilize its positioning and its anionic phenolate state, which provides selectivity for the cationic ferric (Fe^*III*^) form of heme (Moriwaki et al., 2011). The serine in the motif is located in the lip region (Figure 1B, green) on the opposite face and donates a hydrogen bond to heme’s propionate group. A similar mode of heme-binding has been observed in the structures of NEAT domains from other species of bacteria (Honsa et al., 2014). All of the heme-binding NEAT domains in *S. aureus* contain a S/YXXXY motif, but some domains in other bacterial species lack the full complement of motif residues yet can bind heme using distinct axial ligands (Malmirchegini et al., 2014).

The NEAT domains in *S. aureus* have evolved the interesting ability to transiently associate with one another to transfer heme from one NEAT domain to another (Figure 1C; Liu et al., 2008; Villareal et al., 2011; Abe et al., 2012). Heme transfer between the domains occurs very rapidly, ∼70,000-times faster than the rate at which each domain spontaneously releases heme into the solvent. This novel ability led to the intriguing idea that NEAT domains function as heme chaperones within the cell wall, forming a protein wire through which heme is rapidly transferred from the cell surface to the membrane. Liu et al. (2008) were the first to discover that NEAT domains associate with one another to rapidly transfer heme by measuring the rate of heme transfer between the IsdA and IsdC domains using UV-Vis stopped-flow experiments. The extremely rapid kinetics of this process could only be explained by a mechanism in which heme is transferred via an IsdA-IsdC transfer complex. Subsequent studies demonstrated that other NEAT domains within the Isd system also associate with one another to form protein-protein complexes through which heme is rapidly transferred (Zhu et al., 2008b; Villareal et al., 2011; Abe et al., 2012). Transfer between the IsdA and IsdC NEAT domains occurs via an ultra-low affinity complex that forms fleetingly in solution (the IsdA-IsdC transfer complex forms with a K_*D*_ >∼5 mM) (Figure 1C). NMR experiments revealed that the proteins transfer heme via a transiently forming pseudo-symmetrical “handclasp” complex in which the 3_10_-helix of the holo-IsdA donor associates with the loop connecting strands β7 and β8 (β7/β8 loop) in the apo-IsdC acceptor, and vice-versa (Villareal et al., 2011). This basic binding mode was later confirmed using photo-crosslinking and mass spectrometry experiments (Abe et al., 2012). Independently, using molecular docking and atomic structures of isolated NEAT domains, Grigg et al. (2011) also proposed that rapid transfer occurs via a “handclasp” complex. Molecular simulations have provided insight into the IsdA to IsdC heme transfer mechanism, which was modeled to occur via an intermediate in which the tyrosine ligands from each NEAT domain simultaneously form axial linkages to heme’s iron atom such that it is hexacoordinate (Moriwaki et al., 2015). In the intermediate, the metal is coordinated by the primary and secondary tyrosines in the donor and acceptor, respectively, further explaining the functions of these residues in the S/YXXXY motif. Heme flow across the cell wall is driven by a thermodynamic gradient in *S. aureus*, as the heme-binding NEAT domains in the Isd-system exhibit progressively higher affinities for heme the more closely they are positioned to the membrane (Tiedemann and Stillman, 2012; Tiedemann et al., 2012); the affinity increases from IsdB (380 ± 60 nM) (Gaudin et al., 2011), to IsdH (34 ± 8 nM), to IsdA (14 ± 4 nM), to IsdC (6.5 ± 1.4 nM) (Moriwaki et al., 2013). The molecular origins of these affinity differences have been deduced using molecular dynamics simulations with theoretical free energy calculations and *in vitro* isothermal titration calorimetry experiments (Moriwaki et al., 2013).

*In vitro* experiments indicate that IsdC has a unique role in the Isd-system (Figure 1A), accepting heme from upstream NEAT domains in the network and then efficiently transferring it to the IsdE component of the IsdEF importer complex. Transfer to IsdE is thought to occur via a structurally unique IsdC-IsdE transfer complex, since unlike NEAT domains, IsdE adopts a bilobed structural topology in which Met and His residues are the axial ligands such that the iron atom is 6-coordinate (Pluym et al., 2007). Evidence for IsdC-IsdE complex formation comes from mass spectrometry studies that demonstrated that only IsdC efficiently transfers heme to IsdE (Tiedemann and Stillman, 2012) and kinetics measurements that revealed that transfer to IsdE occurs ∼4–10 times faster than the rate at which IsdC releases heme into the solvent (Zhu et al., 2008b). Later studies determined that IsdC transfers heme to IsdE at a rate 2.5 times slower (*k* = 9.6 × 10^–3^ μM^–1^s^–1^) than IsdA to IsdC (2.3 × 10^–2^ μM^–1^s^–1^), indicating that a buildup in IsdC-bound heme is expected at times of heme exposure (Tiedemann et al., 2012). Using crosslinking methods, Abe et al. (2012) obtained direct evidence for complex formation and discovered that a longer β7/β8 strand within IsdC is an important determinant for selective heme transfer to IsdE. IsdC’s location within the cell wall is also presumably critical for its function because it is the only component within the heme transfer network that is attached to the cell wall by SrtB, which is thought to attach IsdC to uncrosslinked glycan chains near the cell membrane (Marraffini and Schneewind, 2005). Moreover, studies of IsdC protein from *Staphylococcus lugdunensis* have shown that it employs a specialized peptidoglycan hydrolase (IsdP, a N-acetylmuramoyl-l-alanine amidase) that affects anchoring of IsdC and consequently the ability of this microbe to use Hb as an iron source (Farrand et al., 2015). Close sequence homologs of IsdC are found in bacterial species within the class Bacilli and are frequently anchored to the cell wall by SrtB enzymes, suggesting that they have similar functions in transferring heme to membrane-associated transporters. *In vitro* evidence for transfer selectivity to ABC-transporter complexes has also been obtained for the IsdC-like proteins in *Streptococcus pyogenes* (called Shp) and *Bacillus anthracis* (Liu and Lei, 2005; Nygaard et al., 2006; Zhu et al., 2008a; Fabian et al., 2009; Balderas et al., 2012).

## Do NEAT Domains in *S. aureus* Form a Protein Wire That Moves Heme Through the Cell Wall?

Based on their spatial positioning within the cell wall and their ability to rapidly transfer heme *in vitro*, it is tempting to speculate that the *S. aureus* NEAT domains don’t simply bind and release heme, but instead physically interact with one another within the cell wall to rapidly transfer heme from the cell surface to the membrane. Why else would the domains have evolved the rare ability to associate and rapidly transfer heme? Although mechanistically attractive, the only direct evidence that NEAT domains interact with one another within the cell wall comes from a cell fractionation and pull-down experiment that demonstrated interactions between IsdA and IsdB (Pishchany et al., 2009). However, these studies need to be revisited, as strong interactions between these proteins have never been demonstrated *in vitro*. Given the presumed structural constraints imposed by the peptidoglycan, it is also unclear how NEAT domains could move within the cell wall to donate and accept heme by forming “handclasp” complexes (Figure 1C; Grigg et al., 2011; Villareal et al., 2011; Abe et al., 2012; Zajdowicz et al., 2012). However, some movement may be possible as the domains are connected to the cell wall by C-terminal polypeptide segments of varying lengths that are presumably unstructured, and the estimated size of the pores that permeate the peptidoglycan are larger than the diameter of the NEAT domains; the diameters of the NEAT domains are 40–50 Å, while pore diameters are estimated to be 50–500 Å depending on the degree of crosslinking and growth stage of the cell (Touhami et al., 2004; Meroueh et al., 2006). Furthermore, NEAT domains can also transfer heme via homotypic interactions (e.g., IsdC to IsdC heme transfer) (Abe et al., 2012), suggesting that a combination of only a few transfer events between like and unlike domains would be sufficient to move heme across the expanse of the cell wall that is 400–1000 Å thick (Geoghegan and Foster, 2017).

## Hb Receptors: NEAT Domains Can Work Together to Bind Hb and Extract Its Heme

IsdB and IsdH (originally called HarA) contain unique Hb-binding NEAT domains that enable them to capture Hb on the microbial surface (Dryla et al., 2003; Torres et al., 2006). In fact, they possess two types of functionally distinct NEAT domains: a canonical heme-binding NEAT domain at their C-termini and one or more Hb-binding domains (Pilpa et al., 2009). The first Hb-binding NEAT domain structures were determined by NMR, with crystal structures of the Hb-bound IsdH-N1 and IsdH-N2 proteins following (Pilpa et al., 2006; Dryla et al., 2007; Krishna Kumar et al., 2011; Dickson et al., 2014; Kumar et al., 2014). More recently, structures of the Hb-binding N1 domain of IsdB in both its apo and Hb-bound forms have been elucidated (Fonner et al., 2014; Bowden et al., 2018). Hb-binding domains are distinguished by the presence of a (F/Y)YH(Y/F) aromatic motif in the lip region and do not contain a S/YXXXY motif. Hb contains α- and β-globin chains that each bind to heme. Structures of isolated IsdB and IsdH NEAT domains free and bound to tetrameric Hb have revealed that residues within their aromatic motifs undergo a disordered-to-ordered transition upon binding, forming a 3_10_ helix that interacts with the A- and E-helices in the globin (Pilpa et al., 2006; Krishna Kumar et al., 2011; Kumar et al., 2014; Macdonald et al., 2019). Heme-binding NEAT domains feature a similarly located binding surface to engage heme. Although the α- and β-globin chains in Hb are structurally similar, subtle variations in the sequence of the aromatic motif enable the IsdH-N1 domain to selectively interact with α-globin, while the IsdH-N2 and IsdB-N1 domains are more promiscuous and bind to both the α- and β-globin subunits of Hb (Dickson et al., 2014). In addition, selectivity for the α chain can be engineered by introducing the appropriate amino acid substitutions into the aromatic motif (Dickson et al., 2015). Interestingly, the residues within the A-helix that form the interface with IsdB and IsdH are important determinants for defining *S. aureus’* host range. In addition, Hb residues at this interface have rapidly changed during primate evolution, consistent with it being the focal point for repeated evolutionary conflicts in the battle for iron during host-pathogen interactions (Pishchany et al., 2010; Choby et al., 2018).

**FIGURE 2:**
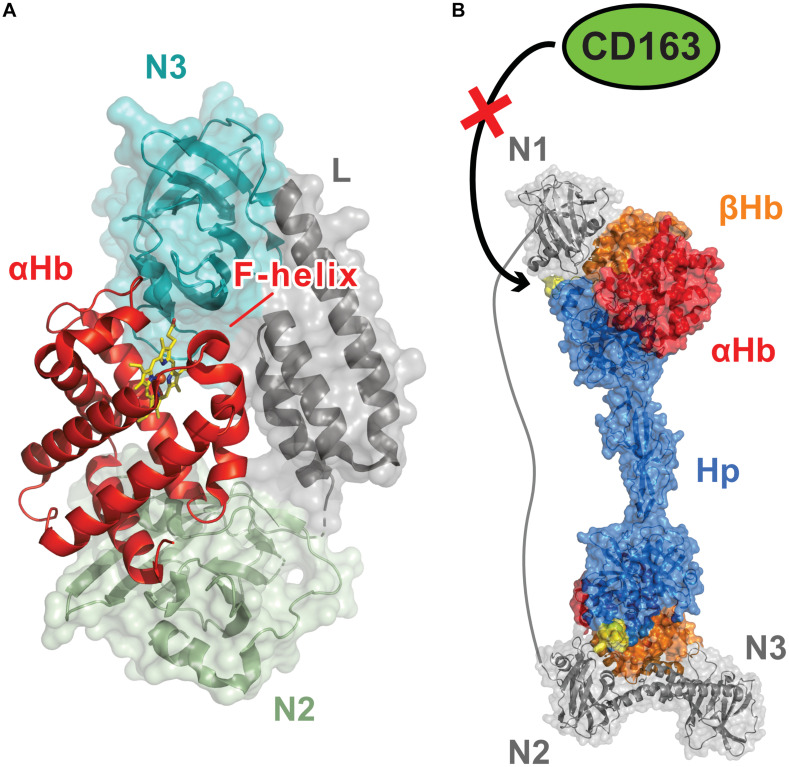
Dedicated Hemoglobin (Hb)-binding NEAT domains enable *S. aureus* to capture Hb and extract its heme molecules. **(A)** Structure of the *S. aureus* IsdH protein bound to Hb [PDB: 4XS0, (Dickson et al., 2015)]. IsdH and IsdB are Hb receptors that extract heme using a conserved tri-domain unit (shown in Figure 1A). Each unit engages a single globin chain and contains a dedicated Hb-binding N-terminal NEAT domain that binds to the globin’s A-helix and a C-terminal NEAT domain then removes the heme molecule via distortion of the Hb F-helix. The tri-domain unit in IsdH contains Hb-binding (N2, green) and heme-binding (N3, teal) NEAT domains, connected by a helical linker domain (L, gray). Synergistic interactions between the NEAT domains trigger heme release from Hb. The IsdB and IsdH tri-domain units bind to Hb in a similar manner and share related primary sequences. **(B)** Model showing how the multiple NEAT domains within the IsdH receptor may prevent haptoglobin (Hp) mediated removal of Hb from the blood, a nutritional immunity process that limits microbial access to iron [PDB: 4WJG, (Stodkilde et al., 2014) 6TB2, (Mikkelsen et al., 2020)] (Figure adapted from Mikkelsen et al., 2020). The tri-domain unit in IsdH is elaborated with a N-terminal Hb-binding NEAT domain (N1). Interactions between N1 and Hb’s β-globin chain in the Hb:Hp complex are thought to disrupt its interactions with the macrophage surface CD163 receptor by occluding the CD163-binding loop of Hp (shown in yellow), thereby preventing the removal of the Hb:Hp complex by receptor mediated endocytosis (Nielsen et al., 2013). IsdB does not contain this accessory N-terminal Hb-binding domain.

The NEAT domains within the IsdB and IsdH Hb receptors work together to actively extract heme from Hb (Spirig et al., 2013). During infections, *S. aureus* encounters the oxidized (ferric) form of Hb (called methemoglobin, metHb), which spontaneously releases heme at a very slow rate (Hargrove et al., 1994). Spirig et al. (2013) discovered that both the IsdB and IsdH proteins contain a conserved tri-domain unit and demonstrated that in IsdH it actively removes heme from metHb. In IsdH, the tri-domain unit (called IsdH^*N2N3*^) is formed by its second (IsdH-N2) and third (IsdH-N3) NEAT domains, which are joined by a helical linker (L) domain (Figure 2A; Spirig et al., 2013). The NEAT domains have distinct functions: IsdH-N2 binds Hb, while the C-terminal IsdH-N3 domain binds to heme (Pilpa et al., 2009). The domains function synergistically and must be part of the same polypeptide in order to effectively induce heme release from Hb, which occurs ∼1,250-fold faster than the rate at which Hb spontaneously releases heme into the solvent (Bowden et al., 2014; Sjodt et al., 2018). Crystal structures of the IsdH^*N2N3*^ complex with Hb reveal that it engages the globin chains within Hb via two distinct binding interfaces (Figure 2A; Dickson et al., 2014, 2015; Mikkelsen et al., 2020). The IsdH-N2 domain interacts with the A- and E-helices of the globin, while the linker and IsdH-N3 domains engage the distally positioned heme pocket on the globin via a Hb-LN3 interface through which heme is transferred. Interestingly, Hb’s heme-contacting F-helix is distorted within the Hb-LN3 interface by two distinct subsites on the receptor that destabilize Hb-heme interactions and form a bridge through which heme moves from Hb to the IsdH-N3 domain (Ellis-Guardiola et al., 2020). NMR studies indicate that prior to engaging Hb, the tri-domain unit adopts an elongated and semi-flexible state in which the linker and IsdH-N3 form a rigid structure that reorient relative to the IsdH-N2 domain (Sjodt et al., 2016). Surprisingly, these inter-domain motions persist in the IsdH^*N2N3*^:Hb complex, enabling the IsdH-N3 domain to transiently engage Hb to remove its heme while remaining tethered to Hb via the IsdH-N2 domain (Ellis-Guardiola et al., 2020). IsdB also extracts heme from Hb using an analogous tri-domain unit that distorts Hb’s F-helix, which is consistent with IsdH and IsdB sharing significant primary sequence homology (64% sequence identity) (Zhu et al., 2008b; Bowden et al., 2014; Figure 1A). Notably, residues in IsdH’s functionally important subsites are conserved in IsdB and both proteins bind and distort Hb in a similar manner, suggesting that they use a generally similar mechanism to extract heme (Fonner et al., 2014; Pishchany et al., 2014; Ellis-Guardiola et al., 2020). However, some differences have been observed, as IsdB is capable of productively extracting heme when its NEAT domains are combined in *trans*, while IsdH must have its full tri-domain unit present as an intact polypeptide to facilitate transfer (Spirig et al., 2013; Bowden et al., 2014). Excitingly, a recent structure of IsdB bound to Hb may have visualized a later step in the heme transfer reaction in which Hb’s Fe^*III*^-His87 bond is broken and the heme molecule is partially transferred to the receptor (Bowden et al., 2018). In the Hb-IsdB crystal structure, the native Hb heme coordination environment (axial coordination by His87 and H_2_O) is disrupted and replaced by a hemichrome exhibiting bis-His metal coordination with the His58 and His89 imidazole rings that are positioned at the edge of Hb’s heme-binding pocket. It is yet to be determined if this hemichrome represents a *bona fide* transfer intermediate or an off-pathway structure that was generated during the prolonged crystallization process.

Increasing evidence suggests that IsdH prolongs microbial access to iron during infections by slowing the rate at which Hb is removed from the blood (Saederup et al., 2016; Mikkelsen et al., 2020). In normal conditions, Hb released from erythrocytes as a result of cellular lysis is bound by haptoglobin (Hp), a highly abundant human glycoprotein that is present in blood plasma. The Hb:Hp complex is then removed from circulation by macrophages via CD163-receptor mediated endocytosis, which limits both the potential damaging effects caused by heme redox chemistry and microbial access to iron (Kristiansen et al., 2001). Unlike IsdB, IsdH elaborates its tri-domain heme extraction unit with an additional N-terminal IsdH-N1 Hb-binding domain (Figure 1A). Initial binding studies using commercially sourced Hp led to the erroneous conclusion that the IsdH-N1 domain could bind to both Hp and Hb (Dryla et al., 2003, 2007; Pilpa et al., 2009). However, more recent studies using purified Hb and Hp proteins have demonstrated that IsdH only interacts with Hb alone or within the Hb:Hp complex (Saederup et al., 2016). The results of these studies suggest that when the intact IsdH receptor captures the Hb:Hp complex on the surface of *S. aureus*, the IsdH-N1 domain may sterically occlude interactions between the complex and the CD163 receptor on macrophages (Nielsen et al., 2013). As a result, *S. aureus* has prolonged access to Hb in blood as macrophages are unable to bind and endocytose the Hb:Hp complex (Figure 2B). Indeed, this may be the primary function of the extra IsdH-N1 domain, as the intact IsdH receptor that contains the additional IsdH-N1 domain is less efficient at scavenging heme from Hb than IsdH’s tri-domain unit. This is presumably because IsdH-N1 binding competes with the extraction unit for binding sites on the globin chains (Mikkelsen et al., 2020). Interestingly, both IsdB and IsdH can’t efficiently extract heme from the Hp:Hb complex and recent results have shown that IsdH is unable to distort Hb’s F-helix when it binds to the Hb:Hp complex (Bowden et al., 2018; Mikkelsen et al., 2020). This suggests that Hp not only removes Hb from the blood, it also actively prevents *S. aureus* from extracting its heme. *S. aureus* mutants lacking *isdB*, but not *isdH*, exhibit reduced virulence in a murine model of abscess formation (Torres et al., 2006). These differences would seem to suggest that in this particular animal model the ability of *S. aureus* to prolong access to Hb using the IsdH protein is less important for pathogenicity than IsdB’s ability to strip heme from Hb.

## NEAT Domains in Other Species of Gram-Positive Bacteria

Genes encoding 343 putative NEAT domains have been identified in over 80 species of bacteria that are almost exclusively found in the Firmicutes phylum (Honsa et al., 2014). A detailed analysis of their sequences suggests that all of these domains will be associated with the cell exterior, either by membrane insertion, sortase-mediated covalent attachment to the cell wall, or secretion, and that nearly half of them will bind to heme (∼48% contain all or most of the residues within the YXXXY motif) (Honsa et al., 2014). In addition to *S. aureus*, NEAT domains have been shown to have a role in heme acquisition in pathogenic *B. anthracis, B. cereus, L. monocytogenes*, and *S. pyogenes* (Bates et al., 2003; Newton et al., 2005; Maresso et al., 2008; Zhu et al., 2008a; Daou et al., 2009; Ouattara et al., 2010; Tarlovsky et al., 2010; Honsa et al., 2011; Balderas et al., 2012). In all instances, these domains function as surface-associated or secreted hemophores, binding heme via the conserved tyrosine linkage. Surprisingly, the genomes of some non-pathogenic soil-dwelling bacteria also encode for domains containing the S/YXXXY motif, which instead of binding heme have been proposed to be involved in chlorophyll uptake. Interestingly, instead of NEAT domains, high G+C Gram-positive bacteria (Actinobacteria) display structurally distinct heme-binding Conserved Region (CR) domains, but it remains unknown whether they function as heme chaperones that rapidly transfer heme.

The results of *in vitro* binding experiments have led to the conclusion that some NEAT domains from *B. anthracis* (IsdX1N and IsdX2N5) and *L. monocytogenes* (Hbp1N) bind to Hb, even though they lack a (F/Y)YH(Y/F) motif (Ekworomadu et al., 2012; Honsa et al., 2013; Malmirchegini et al., 2014). However, recent NMR studies have raised doubts about these findings, because the Hb used in these prior studies was obtained from commercial sources that are now known to contain breakdown products that can lead to erroneous conclusions about Hb binding and Hb-dependent microbial growth (Pishchany et al., 2013; Macdonald et al., 2019). Thus, at present, it appears that only NEAT domains containing a (F/Y)YH(Y/F) motif function as Hb receptors. As NEAT domains are located in extracellular proteins, many might be expected to bind to host proteins other than Hb, or to function as microbial surface components recognizing adhesive matrix molecules. This would seem to make sense, as NEAT domains adopt an immunoglobulin-like fold whose ability to bind to a range of molecules is well documented. Indeed, studies have documented IsdA NEAT domain binding to an array of human proteins (fetuin, asialofetuin, fibrinogen, fibronectin, loricrin, involucrin, and cytokeratin K10) and very recently data demonstrating IsdB interactions with the host protein vitronectin has been reported (Clarke et al., 2004; Pietrocola et al., 2020). However, at present, only structures of NEAT domains bound to either heme or Hb have been determined, so how they recognize these novel binding partners at a molecular level remains to be determined. Finally, as NEAT domains are also widely distributed in many species of non-pathogenic bacteria, these interesting proteins likely perform a range of other functions that have yet to be discovered.

## Author Contributions

All authors listed have made a substantial, direct and intellectual contribution to the work, and approved it for publication.

## Conflict of Interest

The authors declare that the research was conducted in the absence of any commercial or financial relationships that could be construed as a potential conflict of interest.
